# Multivariate Quantitative Outcomes of Periacetabular Osteotomy Using Discrete Element Analysis

**DOI:** 10.1155/aort/1479343

**Published:** 2025-08-22

**Authors:** Victor Grenier, Catherine Ruel, Jean Ruel, Quentin Sercia, Myriam Rioux, Philippe Corbeil, Etienne L. Belzile

**Affiliations:** ^1^Mechanical Engineering Department, Université Laval, Québec, Canada; ^2^Medicine Faculty, Université Laval, Québec, Canada; ^3^Bodycad Laboratories Inc., Québec, Canada; ^4^Department of Kinesiology (Human Movement Analysis and Ergonomics Research Group (GRAME)), Université Laval, Québec, Canada; ^5^Interdisciplinary Research Center on Rehabilitation and Social Integration (CIRRIS), Québec, Canada; ^6^Surgery Department, CHU de Québec-Université Laval, Québec, Canada; ^7^Regenerative Medicine Research Program, CHU de Québec-Université Laval Research Center, Québec, Canada

**Keywords:** cartilage, discrete element analysis, Ganz osteotomy, hip dysplasia, osteoarthritis

## Abstract

**Introduction:** Multiple biomechanical models have been suggested to quantify lower limb joint contact stress distributions, with varying results. Among others, the choice of cartilage morphology and gait loading patterns can significantly affect simulation results. Moreover, there is currently no consensus on simulating the input and output data needed to obtain reliable results and enable a comprehensive analysis.

**Objectives:** The aim of this study was to compare the reliability and clinical relevance of joint contact metrics by calculating pre- and postoperative hip joint contact stress distributions of a dysplastic cohort under various simulation scenarios.

**Methods:** A cohort of 22 dysplastic patients has been treated using periacetabular osteotomy (6-month follow-up). Five radiographic measurements of the acetabular cup were taken from imagery pre- and postoperatively. Eight osteoarthritis-predictive joint stress metrics were computed using discrete element analysis in 6 unique simulation scenarios (2 cartilage models; 3 hip gait loading profiles) pre- and postoperatively.

**Results:** A multivariate analysis of variance confirmed the significant effects of treatment, cartilage model, and loading profile on the computed stress metrics (*p* < 0.01). Also, average- and threshold-based metrics, such as average contact area, average stress, and Maxian overdose, were shown as more reliable indicators of successful surgical treatment than the maximum-based metrics. Finally, correlations between radiographic measurements and stress metrics revealed greater influence of the acetabular index and anterior center-edge angle than the lateral center-edge angle.

**Conclusions:** Average and threshold-based metrics, as well as the acetabular index and anterior center-edge angle, should be of greater interest in future studies regarding hip dysplasia.

**Clinical Significance:** Level 2 (Prospective Study: Therapeutic).

## 1. Introduction

Developmental dysplasia of the hip (DDH) is a genetic condition that affects the normal development of the pelvis. Dysplastic hips are typically shallower and provide insufficient coverage of the femoral head by the acetabulum. This is known to cause greater joint instability, higher articular stresses [1], and the early onset of osteoarthritis (OA) [2, 3].

Radiographic measurements of the acetabular cup, such as the lateral center-edge or acetabular index (AI), are the most important clinical factor for DDH diagnosis and management [4]. Various measurements may be used to quantify the severity of DDH [5–10]. Currently, periacetabular osteotomy (PAO) [11] represents the gold standard for surgical treatment of mature patients with DDH before significant OA development in the affected joint [12]. Although comparative models of stress distribution in the dysplastic and nonpathological hips have been largely studied [13–18], further research is needed to precisely describe the quantitative effects of acetabular realignment procedures on joint stress distributions [19–21].

The most widely used quantitative method to study joint stress is finite element analysis (FEA). However, for large-scale studies, this method remains impractical due to long computation times and the fine-tuning of many sensitive parameters, which can easily hinder convergence reliability. Several studies have shown that discrete element analysis (DEA) has proved to be a reliable and effective alternative to FEA for obtaining data on stress distribution in the joints of the lower limb [16–25].

The metrics used to characterize joint stress distributions can be divided into two broad categories. Some have focused on analyzing singular data, such as maximum stress, in simple static loading scenarios (e.g., single-leg stance) [13, 15, 22–24, 26], suggesting that OA develops in the joint mainly when high stresses occur. Others have focused on more global metrics related to area and time. Metrics such as cumulative stress or average contact area were analyzed in more realistic dynamic loading scenarios (e.g., full gait cycle, running) [13, 21, 27–31]. These metrics further support the idea that the development of OA results from exposure to excessive localized stress over a significant period of time. Although there is a large variety of OA-predictive joint stress metrics, their relative clinical significance remains unknown, as very few studies were found that compared them all using a single cohort of subjects. In addition, there is a trend toward simulating more realistic joint loading scenarios while analyzing them using more comprehensive stress analysis metrics. Moreover, joint stresses are computed with various modeling assumptions. However, there is limited evidence distinguishing whether variance in stress metrics stems from modeling assumptions rather than clinical factors, such as the degree of acetabular coverage. This creates uncertainty as to the scenarios, assumptions, and metrics required to obtain the most reliable results when studying joint stress distribution.

The objective of this study was twofold: first, to assess joint stress metrics during simulated walking in the hip of dysplastic patients before and after PAO, as a function of different modeling parameters: two-hip cartilage models and three-hip joint loading profiles taken from open-source data sets; second, to evaluate the relationship between pre- and postoperative radiographic measurements of acetabular cup orientation and estimated stress metrics, thus quantifying surgical treatment efficiency in light of different modeling parameters.

To achieve this, DEA modeling of the hips of 22 participants pre- and postoperative was used to compute all OA-predictive joint stress metrics known to date. Computed tomography (CT) scan and radiography of the hip were performed pre- and postoperatively to measure acetabular coverage. A sensitivity analysis of the model predictions was performed to highlight the influence of simulation parameters and to identify the most significant and reliable stress metrics associated with the successful treatment of DDH.

## 2. Methods

Clinical Significance: Level 2 (Prospective Study: Therapeutic)

### 2.1. Study Cohort

The study format consisted of a prospective clinical trial of 22 dysplastic patients (16 females, 6 males) who underwent PAO for acetabular positional correction. The inclusion criteria were as follows: (1) a lateral center-edge angle (LCEA)–based DDH diagnosis, (2) more than 16 years of age, and (3) pain in the affected hip. The exclusion criteria were as follows: (1) presenting surgical indications such as neuromuscular hip dysplasia, acetabular retroversion, or coxa profunda; (2) a trauma or surgical history which affects the biomechanics of the dysplastic hip; (3) ipsilateral or contralateral lower limb amputation; (4) gait requiring a walking aid; (5) any prior surgeries that prevented the segmentation of the pelvis. The mean (±SD) age, weight, and height of the cohort were 34.6 (±6.4) years, 72.5 (±14.2) kg, and 1.69 (±0.08) m. Most patients had borderline or mild dysplasia (7/22 and 10/22, respectively) and Grade 0 OA (19/22, Kellgren–Lawrence scale), and one patient was bilaterally affected; therefore, 23 dysplastic hips were studied (see Supporting Information (available here) for cohort detail). Authorization was granted by the Research Ethics Committee of CHU de Québec-Université Laval (2019-07-31).

### 2.2. Imaging Techniques

**Figure 1:**
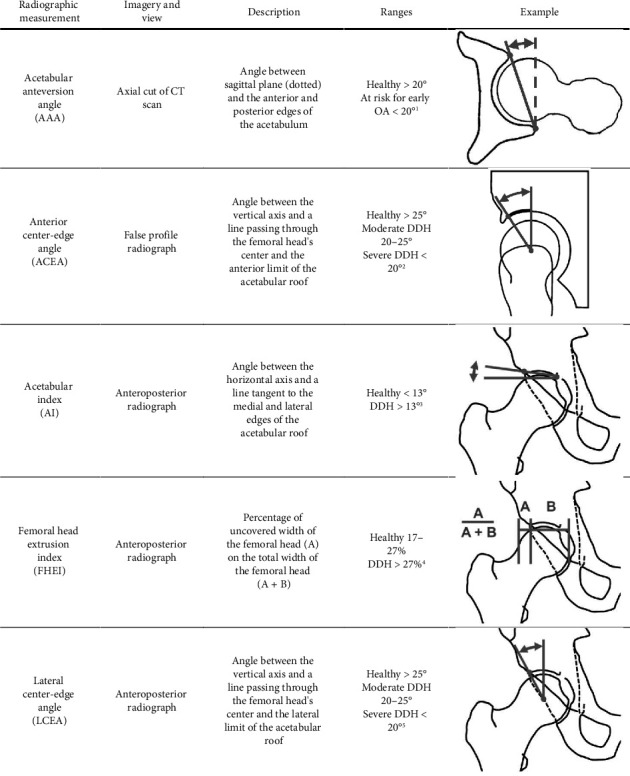
Radiographic measurements of the acetabular cup orientation typically used to characterize congenital hip dysplasia including pathological and nonpathological ranges. Zeng et al. [8], Beltran et al. [38], and Tannast et al. [10]. DDH: developmental dysplasia of the hip.

Planar radiographs and CT were taken for each patient before surgery and at 6-month follow-up. Frontal and sagittal radiographs of the pelvis were taken in a weight-bearing position. Axial CT imaging was acquired in a supine position from the L5 vertebrae to the distal femur (slice thickness: 0.6 mm, interslice distance: 0.7 mm, resolution: 0.48 × 0.48 mm). The following acetabular cup measurements used to characterize DDH were extracted from the imagery: the acetabular anteversion angle (AAA), the anterior center-edge angle (ACEA), the AI, the femoral head extrusion index (FHEI), and the LCEA (Figure 1).

Pelvis and femur volumes were segmented from the CT images using a custom proprietary software (Segmentation Suite, Bodycad Laboratories Inc., QC), resulting in point cloud data and then meshed with an average element size of 0.7 mm (determined with mesh convergence analysis < 2% error). The subchondral lunate surface of the acetabulum, delimited by the convex curvature of the acetabular lip, and the subchondral surface of the femoral head, delimited by the beginning of the femoral neck, were manually selected. The pelvis–femur alignment system proposed by Bergmann et al. [32] was applied to reproduce the neutral weight-bearing position.

### 2.3. Cartilage Volume Generation

**Figure 2:**
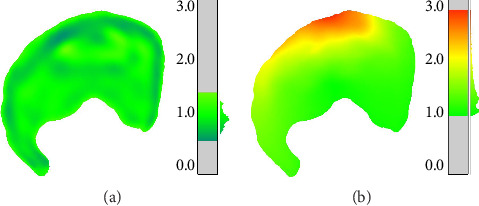
Example of nonpathological (a) and dysplastic (b) acetabular cartilage thickness generation using the “*Shivanna”* and “*Nishii”* models, respectively. On each panel, a thickness mapping of cartilage contact surface mesh and a histogram representation of element thicknesses (mm) for the given mesh are illustrated.

As CT imaging does not allow segmentation of soft tissues such as cartilage and ligaments, the articular cartilage of the femoral head and acetabulum was generated using two algorithms, the “*Shivanna”* model and the “*Nishii”* model. In both algorithms, the cartilage contact surfaces were based on the previously segmented and aligned subchondral surfaces of the femoral head and acetabulum (Figure 2). Both algorithms were implemented using customized MATLAB scripts.

#### 2.3.1. “*Shivanna*” or “*S*” Model Algorithm

The first algorithm, by Shivanna et al. [33], serves to recreate healthy cartilage geometries for the femoral head and acetabulum. The mesh nodes of the femoral head and acetabulum were offset inward 1 mm along their respective radial axes and smoothed toward sphericity 5 times, i.e., by adding the difference between each node's radial distance and the average of neighboring nodes, with a maximum allowable difference of 0.05 mm.

#### 2.3.2. “*Nishii*” or “*N*” Model Algorithm

The second algorithm, based on data from Nishii et al. [34], serves to recreate dysplastic acetabular cartilage, as this population has been observed to display thicker cartilage at the acetabular rim, probably in response to overloading of the specified area. Nishii et al. provide a mapping of average acetabular cartilage thickness data in spherical coordinates for a dysplastic population. A biharmonic spline interpolation was used to generate a continuous thickness distribution. The mesh nodes of the subchondral acetabular surface were offset according to the mapping and re-meshed to create the acetabular cartilage contact surface.

### 2.4. Gait Loading Profiles (GLPs)

Three different GLPs have been used in this study. Each profile is composed of continuous joint reaction forces (JRFs), expressed as body weight percentages, and angular positions (flexion–extension, abduction–adduction, and internal–external rotations) between the proximal femur and pelvis during the stance phase of the gait cycle (in the aligned hip's reference system [32]).

The first profile, taken from Bergmann et al. [32], represents gait based on a cohort with instrumented total hip replacements and is characterized by lower overall JRFs (referred to as the “*Bergmann” or “B”* GLP). The second profile, taken from Harris et al. [35], is based on a dysplastic population and is characterized by higher medially directed JRFs (referred to as the “*Harris” or “H”* GLP). The third profile, taken from Skalshøi et al. [36], is also based on a dysplastic population, but it is characterized by less hip extension, more vertically directed JRFs and overall higher JRFs (referred to as the “*Skalshøi” or “S”* GLP).

**Figure 3:**
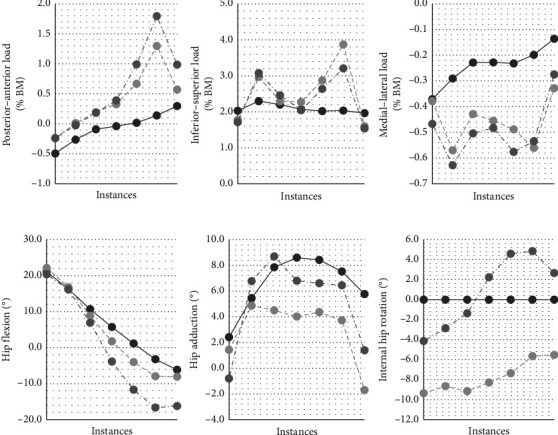
Discretized stance phase of the gait loading profiles (Bergmann (—), Harris (┄), and Skalshøi (- -)). Top: hip joint reaction forces. Bottom: femur–pelvis angular orientation. The Bergmann profile's internal–external rotation was not included in their study and was therefore set to 0°.

For quasistatic simulation purposes, the stance phase of each GLP has been discretized into seven instances. All GLPs were time-normalized by synchronizing their heel strike and push-off peak-loading instances. The 7 evenly time-spaced instances were calculated with the heel strike and push-off as the 2nd and 6th instances, respectively (Figure 3).

### 2.5. DEA

Discrete element analysis (DEA), also known as the bed-of-springs method, was applied to simulate joint loading as described by Abraham et al. [37]. DEA considers the bony subchondral surfaces as rigid and the cartilage as a matrix of springs. Hence, DEA considers only compressive reaction forces caused by contacting surface interpenetration. Cartilage was considered an isotropic linear elastic material with a Young's modulus E of 12 MPa and Poisson's ratio *v* of 0.43. The acetabulum was fixed, and the femoral head had 3 degrees of freedom (DOFs) in translation. The relative angular position between the femur and pelvis was defined by the angles of the given GLP instance. The femoral head was loaded with the JRFs of the given GLP instance. Using a nonlinear least-squares solver (MATLAB *lsqnonlin* function; *Levenberg–Marquardt* algorithm), the femoral head's position was iteratively updated to minimize the squared difference between the applied load and the sum of spring reaction forces. Equilibrium is attained when the residue is less than 1*e*^−6^ N [2]. Inversely, divergence occurs if the simulation fails to reach equilibrium after 400 optimization instances or if the displacement step becomes smaller than 1*e*^−6^ mm. Typically, divergences occurred when the acetabulum has insufficient coverage for the force applied, resulting in subluxation.

### 2.6. Simulation Metrics

All metrics were calculated from the acetabular stress distributions of all 7 instances. If a divergence occurred on one or more instances of a given simulation sequence for a given patient, all results associated with the other instances were omitted from further analysis to prevent altering the definitions of metrics.

Eight metrics have been calculated from the acetabular contact stress distributions pre- and postoperatively (Table 1). According to their definitions, they were categorized into three distinct categories: the maximum, average, and threshold-based metrics. The maximum-based metrics, composed of the *absolute peak stress*, *average peak stress,* and *peak stress–time dose*, measure only the single data point of the highest value of each or all instances. The average-based metrics, composed of the *average contact area* and *average stress*, take into consideration all elements in contact. The threshold-based metrics, composed of the *average supra-threshold area, Maxian overdose,* and *supra-threshold elevated contact area,* each include one or more nonzero thresholds which determine the elements of interest.

### 2.7. Summary of Methods and Statistical Analysis

When paired, the 2 different cartilage models and 3 different GLPs form 6 unique simulation scenarios. Considering 7 discrete instances per GLP, with pre- and postoperative analysis, 84 individual simulations were computed per patient using DEA to obtain joint stress distribution metrics.

Parametric statistics were used as the normality of the distribution was verified with Lilliefors tests. The dependent-samples *t*-test was used to compare pre- and postoperative radiographic measurements. For the joint stress metrics, a multivariate three-way repeated-measures analysis of variance (MANOVA) was used to contrast the factors: treatment (pre- and postoperative), gait loading (*Harris, Skalshøi*, *and Bergmann*), and cartilage model (*Nishii and Shivanna*). The MANOVA allowed to simultaneously analyze all stress metrics as multiple continuous dependent variables. If significance was observed, univariate ANOVAs (as follow-ups to MANOVA) verified which individual variables differ between groups and repeated measures. A Huynh–Feldt correction was applied for violations of sphericity. ES was calculated with partial *η*^2^ for all outcomes, and statistical significance was set a priori at *α* ≤ 0.05.

To perform the sensitivity analysis of the joint stress prediction model, linear correlations between stress metrics and radiographic measurements taken pre- and postoperative were computed for each scenario. The strength of these relationships was estimated with the Pearson correlation coefficient. The number of significant correlations for each pair of measurements was counted and taken as a marker of reliability.

**Figure 4:**
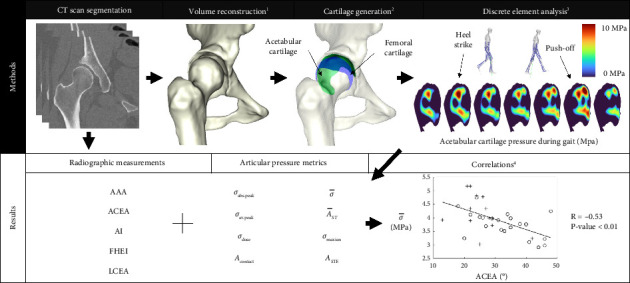
Summary of methods and results. (1) Using Bergmann's alignment method. (2) Using two methods: *Nishii* and *Shivanna.* (3) Using three gait loading profiles: *Bergmann, Harris,* and *Skalshøi.* (4) Calculations were performed after pre- and post-op results acquisition, for all metric and measurement pairs, for all scenarios (total of 240 correlations). Here, the correlation between *ACEA* and *Average Stress* for the *Skalshøi/Nishii* or “SN” scenario (pre-op: “+”, post-op: “o”) is shown.

With regard to the second objective of the study, all twenty-two subjects' pre- and postoperative measurements were included in an exploratory analysis. The analysis was carried out to determine which variables had the most influence on each joint stress metric in the “*Harris-Nishii”* scenario. To do this, multiple regression analysis was done using a forward stepwise method using an F-in of 3.84 and F-out of 2.71 to delineate independent predictors among radiographic measurements of acetabular cup orientation (AAA, ACEA, AI, FHEI, and LCEA) and personal variables (sex, age, height, weight, and acetabular radius estimate). All the statistical tests were conducted with SPSS 25.0 software (IBM Corp., Armonk, NY). A summary of the methods in this study is represented in Figure 4.

## 3. Results

### 3.1. Radiographic Measurements

The average values of all postoperative radiographic measurements (Table 2) corresponded to their target healthy ranges reported in Figure 1. The postoperative success rates for target healthy ranges are 57%, 70%, 100%, 48%, and 74% for the AAA, ACEA, AI, FHEI, and LCEA, respectively (see Supporting Information (available here) for details). With the exception of the AAA, all radiographic measurement variations were significantly different between pre- and postoperative (*p* < 0.01).

### 3.2. Influence of Treatment, Cartilage Model, and GLP

Any diverging simulation results were excluded from the analysis, resulting in uneven sample sizes per scenario. Namely, the sample size of the “Bergmann-Nishii,” “Bergmann-Shivanna,” “Harris-Nishii,” “Harris-Shivanna,” “Skalshøi-Nishii,” and “Skalshøi-Shivanna” scenarios were, respectively, of 17/22, 15/21, 17/22, 15/21, 13/21, and 13/20 (pre/post) converging hips out of 23.

**Figure 5:**
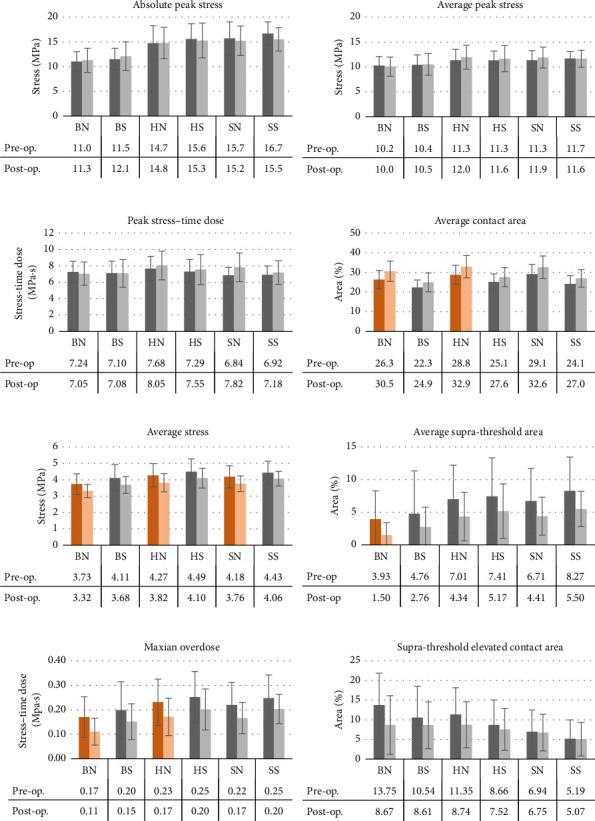
Pre- and postoperative simulation metrics following DEA for all scenarios (“BN,” “BS,” “HN,” “HS,” “SN,” and “SS”). Results represented as mean ± SD. Significant variations of the dependent-samples *t*-test marked in red (*p* value < 0.05).

Figure 5 summarizes the variations in stress metrics pre- and postoperative, according to each scenario. The MANOVA (Table 3) first reveals a significant main effect of treatment, cartilage, and GLP, as well as a significant interaction between treatment and cartilage, and treatment and GLP (*p* < 0.01). Results of the univariate ANOVA showed that treatment caused a significant relative increase in the *contact area* (*p* < 0.001) which was more important in the *Nishii* than in the *Shivanna* scenarios (+14.1% vs. +11.2% for Nishii vs. Shivanna scenario averages). Treatment also caused significant changes in the *average stress, average supra-threshold area,* and *Maxian overdose*, regardless of the cartilage model or GLP tested (*p* < 0.001). Averaged across all scenarios, *average stress*, *average supra-threshold area,* and *Maxian overdose*, relatively decreased by 9.84%, 40.0%, and 24.9%. The influence of cartilage model and loading was also confirmed for a majority of these metrics and are reflected in Figure 5 when visually comparing results between scenarios (e.g. *average stress* is smaller for all “*Nishii”* scenarios vs. their “*Shivanna”* counterparts postoperative). Although nonstatistically significant, the *supra-threshold elevated contact area* seemed influenced by treatment (*p*=0.055). In contrast, treatment caused no significant difference for the maximum-based metrics.

### 3.3. Correlations Between Radiographic Measurements and Simulation Metrics

For all scenarios, a Pearson's weighted correlation has been calculated between the radiographic measurements and simulation metrics (Table 4). Notably, changes in AI correlated with *contact area* (*R* = [−0.39, −0.50], 4/6 scenarios), *average stress* (*R* = [0.38, 0.45, 4/6 scenarios]), and *Maxian overdose* metrics (*R* = [0.36, 0.46], 5/6 scenarios); changes in ACEA correlated with the *average stress* (*R* = [−0.39, −0.53], 3/6 scenarios), *supra-threshold area* (*R* = [−0.33, −0.44], 4/6 scenarios), and *Maxian overdose* (*R* = [−0.41, −0.52], 3/6 scenarios). *R* coefficients for all significant correlations are of moderate strengths, with absolute values between 0.32 and 0.53. The number of significant correlations also varies per scenario.

### 3.4. Predictive Multiple Regression Analysis of Joint Stress Metrics

As the “*Harris-Nishii*” scenario is the pathology-specific parameter set which produced the greater number of significant linear correlations, it was selected for the multiple regression analysis (Table 5). Weight was found to be a common predictor of almost all stress metric estimates. The maximum-based metrics were predicted strictly by weight, whereas all average- and threshold-based metrics had one or more radiographic measurements as significant predictors. Individually, radiographic measurements contributed to predictions with *R*^2^ changes between 0.092 and 0.186. Cumulatively, average- and threshold-based metrics could be estimated with regressions of moderate strengths (Mult. *R*^2^ = [0.301–0.407]).

## 4. Discussion

### 4.1. Summary of Results

As the radiographic measurements are considered the main clinical indicators for DDH severity [4], it was demonstrated that the procedure was successfully performed: with the exception of the AAA, all average measurements were significantly improved from dysplastic to healthy ranges [8, 10, 38] following PAO.

DEA allowed computation of joint pressure distribution during gait in six different scenarios. From these, pressure distributions were extracted eight OA-predictive stress metrics, categorized in three groups: the maximum-, average-, and threshold-based metrics. The MANOVA confirmed an overall significant change in stress metrics following surgical treatment. Univariate ANOVAs further specify that surgical treatment causes significant changes in 4 out of 8 metrics, all of which were average- and threshold-based. In short, our results indicate that the PAO produced a better joint load redistribution across the acetabulum and allowed the avoidance of high stress–time zones throughout the stance phase.

Additionally, the MANOVA confirmed that cartilage model and loading profile caused significant changes in a majority of stress metrics, indicating that simulation outcomes are influenced by the choice of simulation parameters. Other groups have also found joint stress distribution to be affected by reconstruction of bone and cartilage geometries [15, 19, 39, 40] and the choice of GLPs [21, 35, 41].

### 4.2. Not all Metrics Are Equally Reliable

Correlations between stress metrics and radiographic measurements for all scenarios allow to assess the sensitivity of the linear relationships to loading profile and cartilage model. Specifically, when a correlation between a specific metric and radiographic measurement remains significant for many of the scenarios (e.g., AI with *Maxian overdose*: 5 of 6 scenarios), this may indicate a reliable association that is robust to variations in simulation parameters. The most reliable correlations should be of greater clinical interest for PAO planning, as simulation-based analysis and optimization will more often provide an insightful result despite varying simulation parameters.

The average and threshold-based metrics demonstrated significant change following surgery, but not the maximum-based metrics. Also, only a few isolated significant correlations have been noted for the maximum-based metrics, suggesting higher sensitivity to simulation parameters. Finally, our predictive linear regressions indicate that maximum-based metrics do not depend on changes in radiographic measurements, but mainly on patient weight. In comparison, all average and threshold-based metrics are predicted by weight and one or more radiographic measurements. These results suggest that, compared to maximum-based metrics, average and threshold-based metrics could be better indicators of the effect of change in acetabular orientation and therefore better quantitative indicators of successful PAO.

As maximum-based metrics are computed from a singular data point per loading instance, it is reasonable to expect higher sensitivity to localized 3D reconstruction uncertainties. Other groups have found, for example, that a small surface incongruency could lead to a local peak in joint pressure [13, 39, 42]. In comparison, the average and threshold-based metrics represent much more comprehensive results, as they consider the full joint pressure distribution at each loading instant. It is however important to note that threshold-based metrics' sensitivities greatly depend on the chosen threshold values. As the thresholds used in this study have been taken from differing clinical contexts [27, 28, 30], fine-tuning to maximize pre/post-op differentiation for hip dysplasia was not possible.

### 4.3. Predicting Surgical Outcomes

Predictive regressions represent a useful tool to estimate the magnitude of potential joint health improvements. Radiographic measurements, particularly the ACEA and AI, are moderately correlated predictors of stress metrics associated with successful PAO, suggesting these variables should be of greater interest for surgical planning. However, care should be taken not to overcorrect, as an ACEA above 40° may increase the risk of femoroacetabular impingement [43]. Also, weight is shown as a common predictor for almost all stress metrics, meaning weight loss should also be encouraged. Conversely, this also means that weight gain can counteract the benefits of successful PAO. These regressions explain only a portion of the variance observed and could be improved by analyzing additional variables. Improved regressions could help predict when optimal acetabular correction might still be insufficient to significantly improve stress metrics (e.g., for heavier patients). Clinically, this could support the use of other treatment options instead.

### 4.4. Previous Work

A systematic review of the mechanical behavior of dysplastic hips [1] highlights how the majority of past studies almost exclusively focus on peak stress and LCEA. As the current study has shown significant changes for many other stress metrics and radiographic measurements, we propose to expand the scope of analysis in future simulation-based studies of the hip.

Another study [21] found the use of dysplastic gait patterns produced a stronger correlation between LCEA and peak contact stress following PAO, suggesting this pattern should be favored for future studies of dysplastic hips. Although the use of dysplastic GLPs did not systematically produce the strongest correlations in our study, they still demonstrated significant change following PAO. Namely, the *Harris-Nishii* scenario seemed to best reflect changes in the treated hip as it measured significant improvements in stress metrics, which then correlated with changes in multiple radiographic measurements, all while remaining clinically suited to the patient population.

### 4.5. The Limitations of 2D Measurements

The LCEA is currently the most clinically relevant joint health metric for DDH and is often the focus of simulation-based research. When studying DDH and PAO optimization, the LCEA was found to influence peak Von Mises stress [23, 24], peak contact stress [15–17, 21, 44, 45], and contact area [44, 45]. The results of the present study partially reflect those of similar studies, as LCEA was found to correlate with the *contact area*, *average stress,* and *Maxian overdose*, but not with the *peak stress*. In contrast, our results reveal the AI and ACEA as more influential than the LCEA on the simulation metrics. Although rarely examined in comparison with the LCEA, the significant effect of the AI and ACEA has also been noted on other occasions [23, 24, 45].

The greater stabilizing effect of the AI versus LCEA can be explained geometrically. A shallow, laterally “open” acetabulum with an extended rim can have a high LCEA while offering little stability. In comparison, a similarly shallow hip with a shorter acetabular rim (i.e. small LCEA), but a more downward-facing orientation (i.e., small AI) will offer better retention of the femoral head. The “closedness” of the acetabular cup could be more important than the lateral coverage in stabilizing the joint. Similarly, the Nishii cartilage model, which is thicker toward the acetabular rim than the Shivanna cartilage model, could also contribute to this stabilizing effect, possibly explaining why more significant metric improvements occur with this model compared to the other. This study also showed that the ACEA correlated with multiple threshold-based metrics, suggesting that acetabular retroversion may lead to excessive stress on the anterior horn of the acetabulum.

In general, our univariate correlations (|*R*| = [0.32, 0.53]) and the predicted effects of change in stress metrics due to individual acetabular orientation corrections (*R*^2^ = [0.092–0.186]) are weak to moderate, indicating that no single radiographic measurement alone can define optimal joint orientation. Also, these measurements can be affected by factors such as volumetric artifacts and pelvic incidence [46–48]. It therefore seems good practice to consider multiple 2D measurements to define acetabular orientation with better accuracy. More recently, 3D measurement and planning methods have demonstrated improvements over conventional 2D methods for PAO optimization [49, 50] and seem promising moving forward.

### 4.6. Simulations and Divergences Lead to Oversimplifications

Contact simulations are notoriously difficult to perform successfully. Realistic joint contact simulations are even more complex, with high loads, large material deformations, and multiple DOFs. The risk of divergences is further amplified when simulating an inherently unstable joint, such as a dysplastic hip. As a diverging simulation provides no quantitative results to analyze, common practice is to simplify the parameters used to guarantee convergence. However, such simplifications may affect the clinical relevancy of subsequent results. In the specific context of other hip simulation studies, it is frequent to observe at least one of the following:

• Using a single load of low magnitude (e.g., single-leg stance) rather than the full gait cycle including JRF peaks (i.e., heel strike and push-off) [17, 18, 23, 51], reducing the magnitude of stress metrics and the material deformation under loading.
• Using thicker and smoother cartilage geometries instead of more realistic representations [15, 16, 24, 26], minimizing the risk of local pressure peaks and exaggerating contact area.
• Limiting the DOFs of the femoral head to a single vector instead of 3 translational DOFs [23, 24, 44, 45], preventing the femoral head to naturally “sink” to the bottom of the joint, but rather to a predefined position.

### 4.7. Limitations of This Study

Although this study's methods aimed to avoid such simplifications, many limitations remain. Ideally, personalized bone and cartilage geometry would be segmented from imagery (e.g., CTA or high-resolution MRI), and personalized gait analysis would be performed (e.g., motion capture) to obtain the most realistic patient-specific simulation inputs. Furthermore, applying a neutral weight-bearing pelvic alignment may neglect observed interpatient variability in pelvic tilt. As pelvic tilt can affect the radiographic measurements as well as the contact zone in the simulations, this represents a confounding variable in the present study. The use of standing biplanar radiographs could allow for personalized alignment. However, these processes are time-consuming and expensive and the required equipment is not widely available. Whenever such data are not available, chosen simulation parameters should be pathology-specific and results should be paired with a sensitivity analysis. Although DEA has been shown to produce similar results to FEA [25, 40], it does not consider the barreling effect of elements under loading, that is, the bulging or widening of material when compressed which distributes stress transversely across a structure. DEA-computed pressure distributions are therefore characterized by more pronounced local peaks. Furthermore, simulation models could be refined by including additional soft tissue structures, such as the labrum, as its contribution to the load bearing is significant, up to 11% for dysplastic patients [15, 51], and the round ligament of the femoral head, which is known to prevent dislocation of the femoral head. This study also did not include a control group or an analysis of the contralateral limb, which may provide a better understanding of how PAO may restore normal joint behavior in addition to the relative post-op improvements. Finally, gathering additional clinically validated patient reported outcomes data would have allowed to further define surgical success.

## 5. Conclusion

Using DEA, this study assessed the joint stress metrics of the hips of a dysplastic cohort during simulated gait before and after PAO. It then identified the most significant and reliable stress metrics associated with the successful treatment of DDH and evaluated the relationship between pre- and postoperative radiographic measurements of acetabular cup correction and said metrics. Namely, the average and threshold-based metrics have been shown as stronger and more reliable indicators of successful DDH than the more commonly studied maximum-based metrics. Also, the AI and ACEA radiographic measurements have shown a stronger influence than the LCEA on said stress metrics. Finally, the significant influence of cartilage model and GLP choices was highlighted. Greater interest should be given to these radiographic measurements and joint stress metrics for future studies regarding DDH and PAO planning.

## Figures and Tables

**Table 1:** Joint contact stress distribution metrics. Threshold values taken from.

Metric	Equation	Variables
Absolute peak stress (MPa)	*σ*_|.|peak_=max_cycle_(max(*p*_*i*,*j*_))	*p*_*i*,*j*_: elemental stress at instance *j*

Average peak stress (MPa)	*σ*_av.peak_=(1/*t*)∑_*j*=1_^*t*^ max(*p*_*i*,*j*_)	*p*_*i*,*j*_: elemental stress at instance *j*
		*t*: total number of instances

Peak stress–time dose (MPa∗s)	*σ*_dose_=max	*p*_*i*,*j*_: elemental stress at instance *j*
		*t*: total number of instances
		∆*t*: time increment

Average contact area (%)	${\overset{¯}{A}}_{\text{contact}}=\left(100*1/At\right)\sum_{j=1}^{t}\sum_{i=1}^{n}a_{i,j}$	*a*_*i*,*j*_: elemental area if stress > 0 MPa at instance *j*
		*t*: total number of instances
		*n*: total number of elements
		*A*: total acetabular area

Average stress (MPa)	$\overset{¯}{\sigma }=\left(1/t\right)\sum_{j=1}^{t}\left(\sum_{i=1}^{n}p_{i,j}a_{i,j}/\sum_{i=1}^{n}a_{i,j}\right)$	*p*_*i*,*j*_: elemental stress if > 0 MPa at instance *j*
		*a*_*i*,*j*_: elemental area if stress > 0 MPa at instance *j*
		*n*: total amount of elements
		*t*: total number of instances

Average suprathreshold area (%)	${\overset{¯}{A}}_{ST}=\left(100*1/At\right)\sum_{j=1}^{t}\sum_{i=1}^{n}a_{i,j}$	*a*_*i*,*j*_: elemental area if stress > 9.5 MPa [27] at instance *j*
		*t*: total number of instances
		*n*: total number of elements
		*A*: total acetabular area

Maxian overdose (MPa∗s)	*σ*_maxian_=∑_*j*=1_^*t*^(∑_*i*=1_^*n*^*p*_*i*,*j*_*a*_*i*,*j*_/∑_*i*=1_^*n*^*a*_*i*,*j*_)∆*t*	*p*_*i*,*j*_: elemental stress if > 6 MPa [28] at instance *j*
		*a*_*i*,*j*_: elemental area if stress > 6 MPa at instance *j*
		*n*: total amount of elements
		*t*: total number of instances
		∆*t*: time increment

Suprathreshold elevated contact area (%)	*A*_STE_=100∗(∑_*k*=1_^*m*^*a*_*k*_/∑_*i*=1_^*n*^*a*_*i*_)	*a*_*k*_: elemental area if cumulative stress > 5.5 MPa∗s [31]
		*m*: total amount of elements if cumulative stress > 5.5 MPa∗s
		*a*_*i*_: elemental area if stress > 4 MPa at any instant
		*n*: total amount of elements if stress > 4 MPa at any instant

**Table 2:** Radiographic measurements of the acetabular cup before and after periacetabular osteotomy.

Radiological measurement	Mean ± SD		*P* value
	Pre-op.	Post-op.	
AAA	18.0 ± 6.0	20.9 ± 7.2	0.15
ACEA	21.4 ± 12.0	31.8 ± 9.5	**< 0.01**
AI	13.7 ± 8.1	3.4 ± 5.7	**< 0.01**
FHEI	27.3 ± 5.2	16.5 ± 5.6	**< 0.01**
LCEA	17.0 ± 7.4	28.6 ± 5.2	**< 0.01**

*Note:* Bold values indicate *p* value < 0.05.

**Table 3:** Evaluation of influence of cartilage model (*C*), gait loading profiles (*L*), and surgical treatment (*T*) on the simulation joint stress metrics.

		Factors	Treatment	Loading	Cartilage	*L* × *T*	*C* × *T*	*L* × *C*	*L* × *C* × *T*
Multivariate		*p*-value	**< 0.001**	**< 0.001**	**< 0.001**	**0.007**	**< 0.001**	0.278	0.820
		ES	0.70	0.56	0.86	0.38	0.77	0.24	0.15

Univariate									
	Peak stress (max./cycle) (MPa)	*p*-value	0.308	**< 0.001**	**0.047**	0.396	0.795	0.650	0.561
		ES	0.03	0.44	0.10	0.05	0.00	0.02	0.03
	Peak stress (mean/cycle) (MPa)	*p*-value	0.522	**0.031**	0.944	0.443	0.607	0.625	0.847
		ES	0.01	0.17	0.00	0.04	0.01	0.02	0.01
	Peak stress–time dose (sum/cycle) (MPa∗s)	*p*-value	0.131	0.324	0.159	0.118	0.430	0.622	0.833
		ES	0.06	0.06	0.05	0.11	0.02	0.03	0.01
	Contact area (mean/cycle) (% of total)	*p*-value	**< 0.001**	0.272	**< 0.001**	0.949	**< 0.001**	0.719	0.808
		ES	0.36	0.07	0.74	0.00	0.60	0.02	0.01
	Average stress (mean/cycle) (MPa)	*p*-value	**< 0.001**	**0.036**	**< 0.001**	0.951	0.051	0.704	0.548
		ES	0.47	0.16	0.33	0.00	0.10	0.02	0.03
	Suprathreshold area (mean/cycle) (% of total)	*p*-value	**< 0.001**	**0.025**	0.093	0.962	0.669	0.398	0.956
		ES	0.25	0.18	0.07	0.00	0.01	0.05	0.00
	Maxian et al. overdose (sum/cycle) (MPa∗s)	*p*-value	**< 0.001**	**0.050**	**0.004**	0.973	0.052	0.770	0.864
		ES	0.40	0.15	0.20	0.00	0.10	0.01	0.01
	Suprathreshold elevated stress–time dose area (sum/cycle) (% of total)	*p*-value	0.055	0.087	0.164	0.194	0.102	0.838	0.879
		ES	0.09	0.12	0.05	0.08	0.07	0.01	0.01

*Note:* Repeated-measures multivariate ANOVA, CI 95%, bold for *p* values < 0.05.

**Table 4:** Univariate Pearson's weighted correlation coefficients between pressure distribution metrics and radiographic measurements per simulation scenarios (bold for *p* value < 0.05).

Articular pressure metrics	Scenario	Radiographic measurements				
		LCEA	AI	FHEI	ACEA	AAA
Peak stress (max./cycle) (MPa)	BN	0.11	0.06	−0.14	−0.18	0.09
	BS	0.08	0.06	−0.25	−0.10	0.17
	HN	−0.10	0.16	0.01	0.06	0.25
	HS	−0.20	0.30	−0.01	0.04	0.13
	SN	0.02	−0.09	−0.10	−0.20	**0.42**
	SS	−0.09	−0.01	−0.07	0.01	0.18

Peak stress (mean/cycle) (MPa)	BN	−0.02	0.16	−0.01	−0.24	0.02
	BS	−0.02	0.20	−0.17	−0.18	0.12
	HN	0.13	0.00	−0.12	−0.05	0.10
	HS	0.06	0.13	−0.22	0.02	0.11
	SN	0.21	−0.10	−0.26	−0.18	0.28
	SS	0.17	−0.04	−0.32	0.03	0.17

Peak stress–time dose (sum/cycle) (MPa∗s)	BN	−0.04	0.18	0.01	−0.24	−0.01
	BS	−0.08	0.24	−0.10	−0.13	0.07
	HN	0.14	0.01	−0.12	0.01	0.13
	HS	0.04	0.13	−0.18	0.15	0.10
	SN	0.34	−0.13	**−0.39**	0.10	0.19
	SS	0.16	−0.10	−0.27	0.22	0.05

Contact area (mean/cycle) (% of total)	BN	**0.44**	**−0.50**	**−0.32**	0.23	−0.14
	BS	0.25	**−0.35**	−0.21	0.23	−0.21
	HN	**0.38**	**−0.44**	−0.27	0.24	−0.22
	HS	0.21	−0.26	−0.15	0.05	−0.29
	SN	0.33	**−0.39**	−0.17	0.21	−0.29
	SS	0.23	−0.28	−0.21	−0.01	−0.34

Average stress (mean/cycle) (MPa)	BN	**−0.38**	**0.45**	0.30	**−0.40**	0.06
	BS	−0.27	**0.43**	0.14	−0.32	0.06
	HN	**−0.32**	**0.37**	0.27	**−0.39**	0.08
	HS	−0.26	**0.38**	0.11	−0.17	0.12
	SN	−0.22	0.33	0.13	**−0.53**	0.14
	SS	−0.15	0.30	0.04	−0.22	0.08

Suprathreshold area (> 9.5 MPa) (mean/cycle) (% of total)	BN	−0.27	0.28	0.20	**−0.33**	−0.07
	BS	−0.14	0.29	0.01	**−0.37**	0.09
	HN	−0.23	0.30	0.19	**−0.36**	0.00
	HS	−0.19	**0.34**	0.04	−0.21	0.14
	SN	−0.12	0.24	−0.01	**−0.44**	0.23
	SS	−0.08	0.22	−0.03	−0.27	0.14

Maxian et al. overdose (sum/cycle) (MPa∗s)	BN	**−0.37**	**0.46**	**0.32**	**−0.41**	−0.04
	BS	−0.18	**0.37**	0.06	−0.29	0.08
	HN	−0.30	**0.37**	0.26	**−0.42**	0.03
	HS	−0.21	**0.34**	0.06	−0.14	0.11
	SN	−0.19	**0.34**	0.11	**−0.52**	0.11
	SS	−0.08	**0.24**	−0.02	−0.18	0.10

Suprathreshold elevated stress–time dose area (sum/cycle) (% of total)	BN	−0.22	**0.33**	0.22	**−0.37**	−0.30
	BS	−0.05	0.21	−0.05	−0.25	0.01
	HN	−0.12	0.26	0.12	**−0.36**	−0.24
	HS	−0.03	0.22	−0.09	0.09	0.11
	SN	0.08	0.17	−0.09	−0.22	−0.26
	SS	0.04	0.04	−0.11	0.23	−0.13

*Note:* The sum of weights of all preoperative data pairs was equal to the sum of weights of all postoperative data pairs to prevent regression skewing when divergences occurred. *R* coefficients (*p* value < 0.05) are bold.

**Table 5:** Forward stepwise regression summary for joint stress metrics prediction in the “*Harris-Nishii*” scenario.

Predicted joint stress metric	Predictive variable	*B*	Std. err.	*p* value	Step	Mult. *R*^2^	*R* ^2^ change
Peak stress (max./cycle) (MPa)	Intercept	5.103	2.376	**0.038**	—	—	—
	Weight (kg)	0.133	0.032	**< 0.001**	1	0.316	0.316
							= 0.133 ∗ Weight + 5.103

Peak stress (mean/cycle) (MPa)	Intercept	5.379	1.710	**0.003**	—	—	—
	Weight (kg)	0.087	0.023	**< 0.001**	1	0.276	0.276
							= 0.087 ∗ Weight + 5.379

Peak stress–time dose (sum/cycle) (MPa∗s)	Intercept	4.175	1.286	**0.002**	—	—	—
	Weight (kg)	0.051	0.017	**0.006**	1	0.189	0.189
							= 0.051 ∗ Weight + 4.175

Contact area (mean/cycle) (% of total)	Intercept	60.786	15.495	**< 0.001**	—	—	—
	AI (°)	−0.486	0.123	**< 0.001**	1	0.186	0.186
	Weight (kg)	0.202	0.059	**0.002**	2	0.303	0.117
	Height (m)	−24.600	9.910	**0.018**	3	0.407	0.104
							= −0.486∗AI + 0.202 ∗ Weight − 24.6 ∗ Height + 60.786

Average stress (mean/cycle) (MPa)	Intercept	3.300	0.614	**< 0.001**	—	—	—
	Weight (kg)	0.020	0.007	**0.003**	1	0.235	0.235
	ACEA (°)	−0.026	0.010	**0.018**	2	0.347	0.112
							= 0.020 ∗ Weight − 0.026 ∗ ACEA + 3.300

Suprathreshold area (mean/cycle) (% of total)	Intercept	−0.720	4.178	0.864	—	—	—
	Weight (kg)	0.151	0.044	**0.002**	1	0.265	0.265
	ACEA (°)	−0.160	0.070	**0.029**	2	0.357	0.092
							= 0.151 ∗ Weight − 0.160 ∗ ACEA − 0.720

Maxian et al. overdose (sum/cycle) (MPa∗s)	Intercept	0.125	0.082	0.135	—	—	—
	Weight (kg)	0.003	0.001	**0.006**	1	0.215	0.215
	ACEA (°)	−0.004	0.001	**0.010**	2	0.349	0.134
							= 0.003 ∗ Weight − 0.004 ∗ ACEA + 0.125

Suprathreshold elevated stress–time dose area (sum/cycle) (% of total)	Intercept	21.450	3.358	**< 0.001**	—	—	—
	AAA (°)	−0.129	0.045	**0.007**	1	0.145	0.145
	ACEA (°)	−0.286	0.101	**0.008**	2	0.301	0.156
							= −0.129 ∗ AAA − 0.286 ∗ ACEA + 21.450

*Note:* Bold values indicate *p* value < 0.05.

## Data Availability

The data used to support the findings of this study are included within the supporting information file(s).

## Supporting Information

Table S-1: Study cohort demographics.

Table S-2: Radiographic measurements of study cohort pre- and post-PAO.

Table S-3: Joint stress metric data per subject and simulation scenarios. Note: empty cells indicate excluded results due to diverging simulations over the partial or full gait cycle. 0 values indicate convergent simulation.
