# Supplementary material for: Multivariate Quantitative Outcomes of Periacetabular Osteotomy Using Discrete Element Analysis
Source: Adv Orthop. 2025 Aug 22;2025:1479343. doi: 10.1155/aort/1479343 (PMC12396905; doi:10.1155/aort/1479343)
Supplement: Supporting Information — Additional supporting information can be found online in the Supporting Information section. [file 1479343.f1.docx]

Table S-1. Study Cohort Demographics.

| **Subject No.** | **Gender** | **Treated side** | **Age** | **Weight [kg]** | **Height** | **BMI [kg/m2]** | **OA Grade** | **DDH grade*** |
| --- | --- | --- | --- | --- | --- | --- | --- | --- |
|  |  |  | **[yrs]** |  | **[m]** |  |  |  |
| 1 | F | L | 35 | 82 | 1.78 | 25.9 | 0 | moderate |
| 2 | F | R | 37 | 75 | 1.7 | 26 | 0 | borderline |
| 3 | F | R | 41 | 54 | 1.56 | 22.2 | 0 | borderline |
| 4 | F | R/L | 34 | 85 | 1.65 | 31.2 | 0 | borderline |
| 5 | F | R | 28 | 57 | 1.65 | 20.9 | 0 | mild |
| 6 | F | R | 34 | 53 | 1.7 | 18.3 | 0 | borderline |
| 7 | M | L | 41 | 63 | 1.73 | 21 | 0 | severe |
| 8 | F | R | 25 | 79 | 1.85 | 23.1 | 0 | borderline |
| 9 | F | R | 25 | 56 | 1.6 | 21.9 | 0 | mild |
| 10 | M | R | 28 | 70 | 1.83 | 20.9 | 0 | mild |
| 11 | F | R | 33 | 75 | 1.68 | 26.6 | 0 | moderate |
| 12 | F | R | 43 | 77 | 1.55 | 32 | 0 | borderline |
| 13 | F | L | 35 | 66 | 1.73 | 22.1 | 0 | mild |
| 14 | F | L | 44 | 55 | 1.57 | 22.3 | 1 | mild |
| 15 | M | L | 39 | 79 | 1.65 | 29 | 0 | mild |
| 16 | M | L | 22 | 91 | 1.81 | 27.8 | 0 | moderate |
| 17 | M | R | 40 | 83 | 1.68 | 29.4 | 1 | borderline |
| 18 | F | R | 41 | 101 | 1.72 | 34.1 | 0 | mild |
| 19 | F | R | 32 | 66 | 1.68 | 23.4 | 0 | mild |
| 20 | M | R | 36 | 95 | 1.8 | 29.3 | 0 | moderate |
| 21 | F | L | 40 | 54 | 1.63 | 20.3 | 0 | mild |
| 22 | F | R | 28 | 80 | 1.7 | 27.7 | 0 | mild |

*Based on Pre-Op LCEA angle: borderline (20° to <25°), mild (15° to <20°), moderate (0 to <15°) and severe dysplasia (<0°).

Table S-2. Radiographic measurements of study cohort pre- and post-PAO

|  | **LCEA** |  | **AI** |  | **FHEI** |  | **ACEA** |  | **AAA** |  |
| --- | --- | --- | --- | --- | --- | --- | --- | --- | --- | --- |
| **Subject No.** | **Pre** | **Post** | **Pre** | **Post** | **Pre** | **Post** | **Pre** | **Post** | **Pre** | **Post** |
| 1 | 9 | 26 | 23 | 11 | 31 | 15 | 15 | 26 | 18 | 22 |
| 2 | 25 | 31 | 0 | -3 | 22 | 17 | 13 | 20 | 15 | 21 |
| 3 | 20 | 37 | 9 | -5 | 28 | 12 | 25 | 44 | 14 | 22 |
| 4 | 23 | 32 | 12 | 4 | 22 | 14 | 22 | 33 | 18 | 21 |
| 4 | 25 | 28 | 9 | 5 | 20 | 20 | 21 | 35 | 20 | 15 |
| 5 | 19 | 25 | 11 | 7 | 29 | 22 | 25 | 29 | 23 | 19 |
| 6 | 22 | 30 | 3 | -5 | 25 | 17 | 26 | 34 | 27 | 24 |
| 7 | -3 | 19 | 35 | 13 | 37 | 24 | -11 | 17 | 23 | 35 |
| 8 | 28 | 33 | 5 | 2 | 21 | 19 | 22 | 18 | 19 | 14 |
| 9 | 17 | 29 | 10 | -2 | 27 | 16 | 28 | 46 | 18 | 15 |
| 10 | 19 | 25 | 9 | 3 | 28 | 23 | 42 | 41 | 16 | 10 |
| 11 | 4 | 25 | 29 | 10 | 41 | 16 | 10 | 38 | 21 | 29 |
| 12 | 23 | 30 | 7 | 5 | 21 | 13 | 27 | 35 | 13 | 16 |
| 13 | 18 | 38 | 14 | -6 | 27 | 9 | 31 | 46 | 17 | 26 |
| 14 | 15 | 28 | 20 | 8 | 21 | 5 | 11 | 22 | 28 | 28 |
| 15 | 17 | 33 | 13 | -2 | 28 | 9 | 24 | 40 | 21 | 26 |
| 16 | 6 | 17 | 19 | 7 | 31 | 22 | 9 | 31 | 15 | 8 |
| 17 | 25 | 29 | 12 | 6 | 23 | 25 | 29 | 27 | -2 | 18 |
| 18 | 15 | 28 | 14 | 5 | 27 | 19 | 25 | 24 | 16 | 10 |
| 19 | 19 | 24 | 12 | 3 | 26 | 23 | 22 | 32 | 12 | 16 |
| 20 | 13 | 23 | 20 | 12 | 29 | 19 | 4 | 21 | 23 | 29 |
| 21 | 15 | 34 | 17 | 5 | 30 | 7 | 29 | 25 | 15 | 29 |
| 22 | 16 | 34 | 14 | -5 | 31 | 14 | 44 | 48 | 24 | 27 |

Table S-3. Joint Stress Metric Data per subject and simulation scenarios. Note: empty cells indicate excluded results due to diverging simulations over the partial or full gait cycle. 0 values indicate convergent simulation. (Reference abbreviations B:Bergmann, H:Harris, Sk:Skalshoi, N:Nishii, Sh:Shivanna)

| Subject | Gait Loading Profile | Cartilage Generation Model | Absolute Peak Stress [MPa] | | Average Peak Stress [MPa] | | Peak Stress Time Dose [MPa*s] | | Average contact area [%] | | Average Stress [MPa] | | Average Supra Threshold Area [%] | | Maxian Overdose [MPa*s] | | STE contact area [%] | |
| --- | --- | --- | --- | --- | --- | --- | --- | --- | --- | --- | --- | --- | --- | --- | --- | --- | --- | --- |
|  |  |  | Pre | Post | Pre | Post | Pre | Post | Pre | Post | Pre | Post | Pre | Post | Pre | Post | Pre | Post |
| 1 | B | N |  | 10.334 |  | 9.693 |  | 6.963 |  | 0.289 |  | 3.337 |  | 0.716 |  | 0.127 |  | 9.236 |
|  | B | Sh |  | 12.017 |  | 10.692 |  | 7.635 |  | 0.259 |  | 3.624 |  | 2.080 |  | 0.153 |  | 10.868 |
|  | H | N |  | 12.863 |  | 10.212 |  | 6.993 |  | 0.319 |  | 3.695 |  | 2.537 |  | 0.167 |  | 6.841 |
|  | H | Sh |  | 13.382 |  | 11.113 |  | 7.512 |  | 0.288 |  | 3.978 |  | 4.269 |  | 0.187 |  | 7.387 |
|  | Sk | N |  | 15.925 |  | 11.214 |  | 7.133 |  | 0.297 |  | 3.995 |  | 4.630 |  | 0.204 |  | 5.874 |
|  | Sk | Sh |  | 14.895 |  | 11.173 |  | 6.632 |  | 0.270 |  | 4.221 |  | 5.508 |  | 0.225 |  | 2.564 |
| 2 | B | N | 9.310 | 10.462 | 8.685 | 9.044 | 6.212 | 5.716 | 0.322 | 0.379 | 3.389 | 2.740 | 0.000 | 0.161 | 0.098 | 0.041 | 5.264 | 0.867 |
|  | B | Sh | 8.978 | 12.528 | 7.781 | 8.707 | 4.623 | 5.201 | 0.263 | 0.303 | 3.505 | 2.861 | 0.000 | 0.645 | 0.079 | 0.022 | 0.000 | 0.000 |
|  | H | N | 12.584 | 12.051 | 10.338 | 10.955 | 6.473 | 6.951 | 0.342 | 0.387 | 4.053 | 3.241 | 2.078 | 1.370 | 0.186 | 0.087 | 5.663 | 3.358 |
|  | H | Sh | 13.864 | 12.228 | 9.697 | 9.670 | 5.488 | 5.910 | 0.290 | 0.307 | 3.898 | 3.397 | 3.002 | 2.102 | 0.163 | 0.084 | 0.000 | 1.368 |
|  | Sk | N | 16.626 | 14.630 | 10.634 | 11.254 | 5.797 | 6.807 | 0.345 | 0.378 | 3.922 | 3.249 | 2.531 | 1.944 | 0.166 | 0.094 | 1.026 | 3.326 |
|  | Sk | Sh | 18.421 | 14.256 | 11.395 | 10.971 | 6.830 | 6.585 | 0.281 | 0.297 | 3.961 | 3.411 | 5.644 | 3.524 | 0.193 | 0.116 | 1.605 | 3.330 |
| 3 | B | N | 7.682 | 8.503 | 7.336 | 6.998 | 5.193 | 4.987 | 0.365 | 0.386 | 2.583 | 2.698 | 0.000 | 0.000 | 0.046 | 0.025 | 0.000 | 0.000 |
|  | B | Sh | 8.859 | 11.171 | 8.147 | 9.326 | 5.349 | 6.385 | 0.259 | 0.254 | 3.354 | 3.363 | 0.000 | 0.976 | 0.092 | 0.123 | 0.000 | 7.518 |
|  | H | N | 9.522 | 10.509 | 7.713 | 9.882 | 5.404 | 6.754 | 0.408 | 0.433 | 2.933 | 3.005 | 0.026 | 0.512 | 0.070 | 0.066 | 0.000 | 3.605 |
|  | H | Sh | 12.097 | 12.581 | 8.818 | 10.367 | 5.366 | 7.309 | 0.306 | 0.305 | 3.464 | 3.374 | 0.476 | 2.149 | 0.100 | 0.133 | 0.000 | 6.906 |
|  | Sk | N | 10.399 | 10.520 | 7.971 | 8.279 | 4.915 | 5.504 | 0.392 | 0.427 | 3.024 | 2.916 | 0.174 | 0.228 | 0.074 | 0.043 | 0.000 | 0.057 |
|  | Sk | Sh | 13.205 | 13.284 | 9.256 | 10.385 | 5.336 | 6.559 | 0.293 | 0.290 | 3.517 | 3.471 | 1.549 | 2.408 | 0.113 | 0.153 | 0.000 | 3.655 |
| 4-right | B | N | 13.563 | 17.160 | 12.306 | 13.771 | 8.575 | 9.848 | 0.216 | 0.328 | 4.698 | 3.231 | 10.630 | 0.913 | 0.285 | 0.085 | 17.017 | 6.801 |
|  | B | Sh | 16.100 | 20.920 | 14.781 | 16.568 | 10.239 | 11.763 | 0.163 | 0.250 | 5.816 | 4.127 | 18.888 | 2.136 | 0.433 | 0.210 | 19.236 | 7.986 |
|  | H | N | 15.695 | 18.182 | 12.302 | 15.434 | 8.428 | 11.105 | 0.247 | 0.385 | 5.059 | 3.476 | 12.646 | 2.346 | 0.335 | 0.123 | 14.410 | 6.160 |
|  | H | Sh | 18.186 | 21.876 | 13.797 | 18.944 | 9.416 | 13.511 | 0.195 | 0.294 | 5.995 | 4.289 | 19.298 | 3.386 | 0.451 | 0.216 | 17.573 | 5.257 |
|  | Sk | N | 16.381 | 17.849 | 13.089 | 13.851 | 7.522 | 9.945 | 0.245 | 0.371 | 5.166 | 3.515 | 14.893 | 2.759 | 0.344 | 0.135 | 9.894 | 3.980 |
|  | Sk | Sh | 16.580 | 21.633 | 13.868 | 15.675 | 8.273 | 11.176 | 0.197 | 0.281 | 5.890 | 4.362 | 18.789 | 5.010 | 0.445 | 0.224 | 11.103 | 4.091 |
| 4-left | B | N | 12.215 | 9.747 | 11.594 | 9.080 | 8.255 | 6.512 | 0.245 | 0.376 | 4.289 | 3.123 | 8.074 | 0.097 | 0.259 | 0.082 | 18.832 | 7.116 |
|  | B | Sh | 12.659 | 9.944 | 11.791 | 9.409 | 8.070 | 6.136 | 0.168 | 0.287 | 5.574 | 3.714 | 15.429 | 0.586 | 0.423 | 0.159 | 21.400 | 10.168 |
|  | H | N | 14.579 | 11.707 | 11.894 | 9.388 | 8.550 | 6.628 | 0.277 | 0.423 | 4.751 | 3.547 | 10.605 | 1.643 | 0.298 | 0.124 | 12.376 | 5.141 |
|  | H | Sh | 15.582 | 12.571 | 12.048 | 9.459 | 6.985 | 5.713 | 0.208 | 0.328 | 5.569 | 4.029 | 15.363 | 2.295 | 0.403 | 0.180 | 14.600 | 1.107 |
|  | Sk | N | 19.420 | 13.172 | 13.412 | 9.718 | 7.857 | 6.249 | 0.263 | 0.404 | 5.164 | 3.651 | 13.921 | 2.583 | 0.350 | 0.145 | 7.097 | 2.857 |
|  | Sk | Sh | 17.603 | 14.218 | 12.935 | 10.049 | 5.943 | 5.576 | 0.199 | 0.307 | 5.871 | 4.226 | 19.220 | 4.314 | 0.438 | 0.202 | 0.584 | 0.000 |
| 5 | B | N | 10.471 | 10.590 | 10.168 | 10.005 | 7.282 | 7.009 | 0.239 | 0.224 | 3.513 | 3.530 | 2.093 | 1.544 | 0.135 | 0.158 | 12.122 | 16.720 |
|  | B | Sh | 9.305 | 7.604 | 8.701 | 7.068 | 6.078 | 4.791 | 0.227 | 0.228 | 3.321 | 2.985 | 0.000 | 0.000 | 0.093 | 0.034 | 3.672 | 0.000 |
|  | H | N | 12.207 | 12.729 | 10.607 | 12.103 | 7.504 | 8.646 | 0.261 | 0.246 | 4.027 | 4.015 | 4.425 | 5.547 | 0.194 | 0.205 | 8.562 | 14.619 |
|  | H | Sh | 11.211 | 10.584 | 8.458 | 7.270 | 5.543 | 4.613 | 0.251 | 0.242 | 3.730 | 3.449 | 1.131 | 0.561 | 0.137 | 0.066 | 0.166 | 0.000 |
|  | Sk | N |  | 14.440 |  | 12.025 |  | 7.619 |  | 0.240 |  | 3.969 |  | 4.735 |  | 0.192 |  | 6.912 |
|  | Sk | Sh | 14.536 | 12.746 | 9.688 | 8.214 | 5.121 | 5.055 | 0.224 | 0.232 | 4.301 | 3.528 | 6.421 | 1.992 | 0.218 | 0.096 | 0.000 | 0.000 |
| 6 | B | N | 12.432 | 12.055 | 11.346 | 10.838 | 8.061 | 7.603 | 0.218 | 0.249 | 4.170 | 3.617 | 6.438 | 2.307 | 0.229 | 0.175 | 17.586 | 20.094 |
|  | B | Sh | 11.971 | 10.165 | 10.363 | 9.661 | 7.130 | 6.644 | 0.222 | 0.222 | 3.702 | 3.587 | 3.285 | 0.770 | 0.146 | 0.149 | 8.187 | 7.775 |
|  | H | N | 15.893 | 15.406 | 12.390 | 12.834 | 7.674 | 8.817 | 0.234 | 0.263 | 4.737 | 4.221 | 10.076 | 7.570 | 0.299 | 0.245 | 9.770 | 17.089 |
|  | H | Sh | 15.083 | 14.433 | 11.310 | 10.238 | 6.535 | 6.911 | 0.232 | 0.259 | 4.349 | 3.852 | 5.863 | 1.993 | 0.241 | 0.171 | 2.780 | 3.522 |
|  | Sk | N | 23.365 | 16.150 | 14.435 | 13.202 | 6.566 | 8.638 | 0.226 | 0.259 | 4.775 | 4.128 | 11.209 | 7.646 | 0.300 | 0.223 | 4.045 | 11.325 |
|  | Sk | Sh | 21.911 | 17.342 | 13.027 | 11.955 | 7.119 | 8.291 | 0.223 | 0.239 | 4.449 | 3.996 | 9.088 | 6.713 | 0.239 | 0.212 | 2.070 | 8.997 |
| 7 | B | N |  |  |  |  |  |  |  |  |  |  |  |  |  |  |  |  |
|  | B | Sh |  | 15.047 |  | 13.685 |  | 9.677 |  | 0.161 |  | 4.705 |  | 11.855 |  | 0.291 |  | 16.947 |
|  | H | N |  |  |  |  |  |  |  |  |  |  |  |  |  |  |  |  |
|  | H | Sh |  |  |  |  |  |  |  |  |  |  |  |  |  |  |  |  |
|  | Sk | N |  |  |  |  |  |  |  |  |  |  |  |  |  |  |  |  |
|  | Sk | Sh |  |  |  |  |  |  |  |  |  |  |  |  |  |  |  |  |
| 8 | B | N | 11.665 | 11.770 | 11.174 | 11.049 | 7.756 | 7.949 | 0.303 | 0.287 | 3.654 | 3.871 | 3.915 | 5.363 | 0.158 | 0.187 | 15.757 | 22.331 |
|  | B | Sh | 13.566 | 11.304 | 12.139 | 10.672 | 7.658 | 7.603 | 0.231 | 0.208 | 3.971 | 4.273 | 5.748 | 4.670 | 0.220 | 0.251 | 19.426 | 21.274 |
|  | H | N | 16.438 | 15.257 | 12.751 | 12.916 | 8.461 | 9.053 | 0.307 | 0.299 | 4.651 | 4.748 | 10.333 | 12.338 | 0.279 | 0.297 | 16.392 | 21.647 |
|  | H | Sh | 16.015 | 16.111 | 12.687 | 12.762 | 8.597 | 9.068 | 0.267 | 0.262 | 4.317 | 4.364 | 7.419 | 8.239 | 0.238 | 0.259 | 12.551 | 15.338 |
|  | Sk | N | 16.828 | 14.002 | 12.660 | 11.700 | 7.494 | 8.401 | 0.310 | 0.306 | 4.338 | 4.424 | 7.692 | 6.816 | 0.234 | 0.255 | 12.145 | 15.085 |
|  | Sk | Sh | 15.648 | 14.274 | 12.714 | 11.935 | 8.046 | 7.528 | 0.263 | 0.259 | 4.160 | 4.206 | 7.174 | 7.210 | 0.224 | 0.237 | 6.048 | 12.146 |
| 9 | B | N | 10.152 | 8.065 | 9.566 | 7.703 | 6.870 | 5.462 | 0.249 | 0.298 | 3.430 | 2.833 | 0.622 | 0.000 | 0.137 | 0.038 | 11.780 | 0.000 |
|  | B | Sh | 10.050 | 12.941 | 9.404 | 10.292 | 6.514 | 7.289 | 0.221 | 0.212 | 3.683 | 3.498 | 0.978 | 2.981 | 0.157 | 0.118 | 9.774 | 9.952 |
|  | H | N | 13.803 | 10.433 | 10.291 | 9.012 | 7.341 | 5.646 | 0.263 | 0.305 | 3.982 | 3.389 | 3.772 | 0.531 | 0.196 | 0.103 | 9.180 | 0.480 |
|  | H | Sh | 13.349 | 14.073 | 9.944 | 10.803 | 6.440 | 7.239 | 0.234 | 0.234 | 4.237 | 4.022 | 4.828 | 4.985 | 0.217 | 0.189 | 3.220 | 5.657 |
|  | Sk | N | 13.310 | 10.174 | 10.528 | 8.913 | 7.134 | 5.055 | 0.264 | 0.319 | 3.999 | 3.221 | 4.550 | 0.107 | 0.203 | 0.086 | 6.507 | 0.000 |
|  | Sk | Sh | 14.035 | 14.293 | 10.336 | 10.749 | 6.054 | 6.414 | 0.238 | 0.242 | 4.201 | 3.842 | 5.181 | 4.103 | 0.211 | 0.167 | 1.922 | 2.429 |
| 10 | B | N | 6.894 | 10.241 | 6.388 | 9.283 | 4.292 | 6.633 | 0.264 | 0.290 | 2.789 | 2.726 | 0.000 | 0.135 | 0.006 | 0.044 | 0.000 | 4.166 |
|  | B | Sh | 11.002 | 9.555 | 9.907 | 8.963 | 6.911 | 5.989 | 0.194 | 0.208 | 3.565 | 3.522 | 1.056 | 0.009 | 0.122 | 0.121 | 7.660 | 2.814 |
|  | H | N | 11.983 | 13.503 | 7.724 | 10.322 | 4.896 | 7.227 | 0.298 | 0.328 | 3.210 | 3.134 | 0.745 | 1.300 | 0.059 | 0.080 | 0.000 | 4.488 |
|  | H | Sh | 16.029 | 16.561 | 12.350 | 11.782 | 8.559 | 7.900 | 0.200 | 0.214 | 4.238 | 4.190 | 7.189 | 4.673 | 0.236 | 0.229 | 13.893 | 12.923 |
|  | Sk | N | 13.710 | 13.791 | 8.719 | 11.053 | 6.063 | 7.906 | 0.287 | 0.320 | 3.250 | 3.108 | 1.372 | 1.724 | 0.068 | 0.081 | 0.783 | 4.701 |
|  | Sk | Sh | 17.636 | 17.216 | 12.650 | 12.879 | 8.015 | 8.771 | 0.192 | 0.207 | 4.353 | 4.279 | 8.690 | 6.618 | 0.257 | 0.238 | 14.841 | 11.206 |
| 11 | B | N |  | 10.577 |  | 9.716 |  | 6.986 |  | 0.277 |  | 3.316 |  | 0.668 |  | 0.105 |  | 7.169 |
|  | B | Sh |  | 13.278 |  | 11.458 |  | 7.052 |  | 0.243 |  | 3.706 |  | 2.798 |  | 0.157 |  | 7.316 |
|  | H | N |  | 12.661 |  | 9.984 |  | 6.755 |  | 0.297 |  | 3.810 |  | 1.484 |  | 0.146 |  | 3.758 |
|  | H | Sh |  | 18.457 |  | 13.248 |  | 8.410 |  | 0.251 |  | 4.327 |  | 5.122 |  | 0.223 |  | 6.962 |
|  | Sk | N |  | 11.611 |  | 9.630 |  | 6.073 |  | 0.294 |  | 3.771 |  | 1.776 |  | 0.163 |  | 1.124 |
|  | Sk | Sh |  | 16.259 |  | 12.228 |  | 7.445 |  | 0.257 |  | 4.153 |  | 5.787 |  | 0.225 |  | 2.617 |
| 12 | B | N | 13.394 | 11.307 | 12.344 | 10.681 | 8.824 | 7.680 | 0.346 | 0.370 | 3.620 | 3.423 | 7.827 | 0.904 | 0.190 | 0.143 | 29.479 | 19.921 |
|  | B | Sh | 11.873 | 8.771 | 10.406 | 8.414 | 7.301 | 5.889 | 0.302 | 0.333 | 3.793 | 3.347 | 2.330 | 0.000 | 0.155 | 0.069 | 10.492 | 2.466 |
|  | H | N | 13.905 | 15.881 | 12.622 | 14.847 | 8.669 | 10.662 | 0.377 | 0.380 | 4.168 | 4.062 | 8.959 | 5.269 | 0.235 | 0.209 | 20.380 | 14.771 |
|  | H | Sh | 13.671 | 12.839 | 10.068 | 9.486 | 6.269 | 6.197 | 0.342 | 0.366 | 4.123 | 3.722 | 3.537 | 1.644 | 0.205 | 0.139 | 6.483 | 5.159 |
|  | Sk | N | 14.259 | 14.198 | 12.151 | 13.072 | 8.122 | 9.387 | 0.363 | 0.383 | 4.332 | 3.979 | 8.230 | 4.075 | 0.246 | 0.195 | 15.063 | 10.630 |
|  | Sk | Sh | 17.052 | 15.316 | 11.374 | 10.837 | 6.928 | 6.593 | 0.338 | 0.344 | 4.125 | 3.868 | 6.160 | 4.356 | 0.208 | 0.176 | 7.614 | 8.304 |
| 13 | B | N | 10.063 | 9.893 | 9.217 | 8.657 | 6.552 | 5.650 | 0.217 | 0.268 | 3.263 | 2.664 | 0.137 | 0.083 | 0.126 | 0.065 | 12.248 | 0.714 |
|  | B | Sh | 9.823 | 11.902 | 9.441 | 10.563 | 6.751 | 7.422 | 0.201 | 0.218 | 3.467 | 3.254 | 0.351 | 2.768 | 0.129 | 0.136 | 8.575 | 13.932 |
|  | H | N | 14.976 | 14.772 | 10.825 | 10.559 | 7.512 | 7.392 | 0.247 | 0.294 | 3.567 | 3.006 | 3.558 | 1.615 | 0.161 | 0.085 | 13.841 | 5.638 |
|  | H | Sh | 14.669 | 16.265 | 10.399 | 12.092 | 7.087 | 8.087 | 0.236 | 0.229 | 3.660 | 3.766 | 3.162 | 5.741 | 0.157 | 0.185 | 9.299 | 15.038 |
|  | Sk | N |  | 16.324 |  | 11.410 |  | 7.874 |  | 0.287 |  | 2.980 |  | 2.944 |  | 0.105 |  | 7.064 |
|  | Sk | Sh | 17.589 | 17.252 | 11.774 | 12.685 | 8.088 | 7.961 | 0.222 | 0.218 | 3.872 | 3.838 | 5.357 | 6.531 | 0.186 | 0.193 | 10.182 | 11.059 |
| 14 | B | N |  | 10.120 |  | 9.432 |  | 6.713 |  | 0.275 |  | 3.330 |  | 0.410 |  | 0.103 |  | 7.171 |
|  | B | Sh |  |  |  |  |  |  |  |  |  |  |  |  |  |  |  |  |
|  | H | N |  | 15.311 |  | 11.061 |  | 7.839 |  | 0.291 |  | 3.780 |  | 4.708 |  | 0.165 |  | 10.173 |
|  | H | Sh |  |  |  |  |  |  |  |  |  |  |  |  |  |  |  |  |
|  | Sk | N |  | 16.990 |  | 12.094 |  | 8.436 |  | 0.267 |  | 4.114 |  | 9.408 |  | 0.214 |  | 10.792 |
|  | Sk | Sh |  |  |  |  |  |  |  |  |  |  |  |  |  |  |  |  |
| 15 | B | N | 11.318 | 15.199 | 10.197 | 12.689 | 7.131 | 9.088 | 0.241 | 0.318 | 4.224 | 3.156 | 5.400 | 2.256 | 0.236 | 0.068 | 14.793 | 6.496 |
|  | B | Sh | 12.059 | 10.988 | 10.892 | 9.749 | 7.650 | 5.576 | 0.218 | 0.260 | 4.262 | 3.405 | 6.229 | 0.844 | 0.212 | 0.108 | 12.693 | 0.436 |
|  | H | N | 15.939 | 20.335 | 12.250 | 15.173 | 8.375 | 10.817 | 0.266 | 0.328 | 4.790 | 3.725 | 10.778 | 4.477 | 0.313 | 0.128 | 19.349 | 6.275 |
|  | H | Sh | 15.271 | 16.557 | 10.893 | 12.060 | 7.270 | 7.716 | 0.248 | 0.268 | 4.641 | 4.033 | 7.822 | 4.181 | 0.270 | 0.193 | 11.547 | 6.010 |
|  | Sk | N | 16.974 | 21.316 | 12.518 | 15.937 | 7.125 | 11.276 | 0.260 | 0.321 | 4.820 | 3.760 | 12.388 | 6.139 | 0.314 | 0.140 | 8.358 | 6.528 |
|  | Sk | Sh | 14.362 | 17.567 | 10.804 | 12.702 | 6.664 | 7.910 | 0.241 | 0.267 | 4.690 | 3.954 | 9.316 | 5.340 | 0.281 | 0.181 | 4.290 | 4.166 |
| 16 | B | N |  | 12.490 |  | 11.568 |  | 8.294 |  | 0.315 |  | 3.620 |  | 5.294 |  | 0.165 |  | 16.241 |
|  | B | Sh |  | 16.278 |  | 13.652 |  | 9.736 |  | 0.286 |  | 3.782 |  | 6.354 |  | 0.175 |  | 12.854 |
|  | H | N |  | 17.701 |  | 12.766 |  | 8.226 |  | 0.380 |  | 3.695 |  | 6.210 |  | 0.170 |  | 13.986 |
|  | H | Sh |  | 19.209 |  | 13.139 |  | 9.034 |  | 0.329 |  | 4.039 |  | 6.819 |  | 0.196 |  | 8.050 |
|  | Sk | N |  | 19.472 |  | 13.955 |  | 8.978 |  | 0.362 |  | 3.908 |  | 7.321 |  | 0.197 |  | 13.873 |
|  | Sk | Sh |  | 17.895 |  | 13.299 |  | 8.686 |  | 0.320 |  | 4.173 |  | 8.438 |  | 0.220 |  | 8.868 |
| 17 | B | N | 11.142 | 15.238 | 10.135 | 13.915 | 7.272 | 9.994 | 0.268 | 0.304 | 3.449 | 3.648 | 1.978 | 2.036 | 0.178 | 0.182 | 23.814 | 19.766 |
|  | B | Sh |  | 9.989 |  | 8.432 |  | 5.272 |  | 0.281 |  | 2.865 |  | 0.052 |  | 0.058 |  | 0.000 |
|  | H | N | 12.938 | 17.569 | 11.873 | 16.099 | 7.718 | 11.509 | 0.291 | 0.340 | 3.913 | 3.843 | 6.262 | 4.247 | 0.215 | 0.195 | 20.534 | 15.270 |
|  | H | Sh |  | 10.623 |  | 8.702 |  | 5.422 |  | 0.325 |  | 2.975 |  | 0.313 |  | 0.061 |  | 0.000 |
|  | Sk | N | 11.741 | 17.742 | 10.811 | 14.366 | 7.055 | 10.153 | 0.308 | 0.346 | 3.714 | 3.695 | 3.858 | 3.737 | 0.199 | 0.184 | 16.925 | 13.840 |
|  | Sk | Sh |  | 11.227 |  | 9.385 |  | 5.487 |  | 0.301 |  | 3.185 |  | 0.403 |  | 0.093 |  | 0.000 |
| 18 | B | N | 13.891 | 14.035 | 13.228 | 12.229 | 9.476 | 7.901 | 0.229 | 0.354 | 4.929 | 4.092 | 14.501 | 6.369 | 0.312 | 0.211 | 25.578 | 13.358 |
|  | B | Sh | 15.447 | 11.819 | 13.824 | 11.087 | 9.733 | 7.163 | 0.209 | 0.292 | 5.233 | 4.436 | 16.199 | 4.565 | 0.350 | 0.254 | 26.557 | 14.489 |
|  | H | N | 19.757 | 19.421 | 14.186 | 16.482 | 9.853 | 8.645 | 0.251 | 0.362 | 5.715 | 5.207 | 18.846 | 13.768 | 0.420 | 0.355 | 21.074 | 15.758 |
|  | H | Sh | 22.164 | 18.481 | 15.144 | 15.327 | 9.923 | 7.940 | 0.241 | 0.336 | 5.761 | 5.130 | 18.022 | 9.871 | 0.423 | 0.339 | 19.076 | 6.958 |
|  | Sk | N |  | 16.116 |  | 14.063 |  | 7.623 |  | 0.378 |  | 4.742 |  | 9.353 |  | 0.287 |  | 8.705 |
|  | Sk | Sh |  | 14.648 |  | 11.813 |  | 6.387 |  | 0.331 |  | 4.892 |  | 8.141 |  | 0.300 |  | 2.365 |
| 19 | B | N | 10.227 | 7.250 | 9.499 | 6.730 | 6.745 | 4.750 | 0.237 | 0.297 | 3.574 | 3.101 | 0.818 | 0.000 | 0.128 | 0.056 | 8.834 | 0.000 |
|  | B | Sh |  |  |  |  |  |  |  |  |  |  |  |  |  |  |  |  |
|  | H | N | 11.439 | 9.375 | 8.847 | 7.720 | 6.282 | 4.821 | 0.277 | 0.324 | 3.864 | 3.575 | 2.004 | 0.000 | 0.157 | 0.126 | 3.621 | 0.000 |
|  | H | Sh |  | 11.228 |  | 9.429 |  | 6.203 |  | 0.252 |  | 4.144 |  | 3.176 |  | 0.206 |  | 4.443 |
|  | Sk | N | 14.272 | 10.935 | 9.129 | 7.941 | 5.665 | 4.286 | 0.274 | 0.324 | 3.896 | 3.562 | 2.538 | 1.221 | 0.161 | 0.112 | 0.597 | 0.000 |
|  | Sk | Sh |  | 12.910 |  | 9.924 |  | 5.463 |  | 0.252 |  | 4.105 |  | 3.475 |  | 0.211 |  | 0.000 |
| 20 | B | N |  | 10.348 |  | 8.681 |  | 5.863 |  | 0.276 |  | 3.802 |  | 0.854 |  | 0.143 |  | 2.445 |
|  | B | Sh |  | 11.833 |  | 10.295 |  | 6.457 |  | 0.233 |  | 4.308 |  | 3.713 |  | 0.218 |  | 9.275 |
|  | H | N |  | 19.842 |  | 12.015 |  | 7.169 |  | 0.272 |  | 4.717 |  | 9.011 |  | 0.279 |  | 8.004 |
|  | H | Sh |  | 22.844 |  | 14.420 |  | 8.595 |  | 0.219 |  | 5.585 |  | 18.233 |  | 0.393 |  | 17.110 |
|  | Sk | N |  |  |  |  |  |  |  |  |  |  |  |  |  |  |  |  |
|  | Sk | Sh |  |  |  |  |  |  |  |  |  |  |  |  |  |  |  |  |
| 21 | B | N | 9.293 | 13.001 | 9.050 | 10.427 | 6.507 | 7.156 | 0.221 | 0.190 | 3.540 | 3.532 | 0.000 | 2.898 | 0.134 | 0.128 | 9.789 | 7.904 |
|  | B | Sh | 9.848 | 14.392 | 9.303 | 12.604 | 6.598 | 8.543 | 0.197 | 0.157 | 3.877 | 4.192 | 0.266 | 7.967 | 0.147 | 0.231 | 7.373 | 13.901 |
|  | H | N | 13.168 | 13.885 | 10.018 | 11.720 | 6.587 | 7.156 | 0.240 | 0.213 | 4.081 | 3.865 | 2.500 | 5.318 | 0.208 | 0.188 | 7.485 | 8.840 |
|  | H | Sh | 15.018 | 17.043 | 10.586 | 13.803 | 6.745 | 8.528 | 0.224 | 0.174 | 4.231 | 4.646 | 3.794 | 11.124 | 0.220 | 0.287 | 7.748 | 14.067 |
|  | Sk | N | 16.391 | 15.383 | 11.432 | 12.894 | 7.571 | 7.781 | 0.244 | 0.200 | 3.998 | 4.025 | 3.899 | 8.522 | 0.198 | 0.215 | 7.791 | 8.181 |
|  | Sk | Sh | 18.306 | 16.961 | 12.063 | 14.208 | 7.587 | 7.729 | 0.227 | 0.169 | 4.184 | 4.637 | 4.961 | 12.816 | 0.207 | 0.286 | 7.222 | 11.742 |
| 22 | B | N | 13.131 | 9.461 | 11.962 | 8.892 | 8.096 | 6.273 | 0.292 | 0.368 | 4.342 | 3.542 | 4.461 | 0.000 | 0.238 | 0.103 | 10.833 | 2.294 |
|  | B | Sh | 10.800 | 11.643 | 9.118 | 9.164 | 5.959 | 6.394 | 0.274 | 0.331 | 4.458 | 3.741 | 0.690 | 2.180 | 0.224 | 0.150 | 3.126 | 10.841 |
|  | H | N | 25.118 | 16.069 | 16.195 | 11.522 | 10.795 | 8.218 | 0.308 | 0.374 | 5.081 | 4.222 | 11.483 | 3.667 | 0.305 | 0.226 | 10.346 | 6.297 |
|  | H | Sh | 21.712 | 12.606 | 13.110 | 10.526 | 9.143 | 6.826 | 0.291 | 0.313 | 5.128 | 4.800 | 10.302 | 7.640 | 0.316 | 0.301 | 10.982 | 14.611 |
|  | Sk | N |  | 18.863 |  | 12.770 |  | 9.141 |  | 0.370 |  | 4.238 |  | 4.854 |  | 0.223 |  | 6.860 |
|  | Sk | Sh |  | 15.825 |  | 11.543 |  | 7.311 |  | 0.310 |  | 4.816 |  | 7.260 |  | 0.308 |  | 3.929 |
